# Causal relationship between plasma metabolites and chronic regional pain: a Mendelian randomization study

**DOI:** 10.1016/j.metop.2026.100456

**Published:** 2026-03-07

**Authors:** Yanwen Li, Muzi Li, Kang Peng, Wei Zhang, Liling Guo, Long Chen

**Affiliations:** a Department of Neurosurgery, The First Affiliated Hospital of Anhui Medical University, Hefei, China; b Department of Neurosurgery, The Second Xiangya Hospital, Central South University, Changsha, Hunan, China; c Department of Neurosurgery, Xiangya Hospital, Central South University, Changsha, China; d Department of General Medicine, The First Affiliated Hospital of USTC, Division of Life Sciences and Medicine, University of Science and Technology of China, Hefei, China

**Keywords:** Plasma metabolites, Chronic pain, Mendelian randomization, SNPs, IVs

## Abstract

**Background:**

Chronic pain imposes an enormous economic and personal health burden worldwide, with more than one-third of the population affected. However, no studies have systematically analyzed the potential role of plasma metabolites in chronic pain. This study aimed to perform an exploratory, hypothesis-generating Mendelian randomization (MR) analysis to identify candidate metabolite–pain relationships.

**Methods:**

Pooled genome-wide association study (GWAS) data for 1400 plasma metabolites from previous research was used as exposures and genetic data from the UK Biobank related to eight chronic regional pains were used as outcomes, including headache, facial pain, neck and shoulder pain, back pain, hip pain, stomachache, knee pain, and general pain. Instrumental variables (IVs) for metabolites were selected at a significance threshold of *p* < 1 × 10^−5^ to ensure sufficient statistical power, with an F-statistic >10 to minimize weak instrument bias. Causal associations between genetically predicted plasma metabolites and chronic regional pain were analyzed using the inverse variance weighting (IVW) method as the primary MR approach. Horizontal pleiotropy tests and sensitivity analyses were performed for each pain phenotype using MR-PRESSO and leave-one-out analyses. In addition, four complementary MR methods-weighted median, sample mode, weighted mode, and MR Egger-were applied to strengthen robustness of the findings. Finally, reverse MR analyses were performed to further refine the results.

**Results:**

MR analyses identified 122 plasma metabolites associated with eight chronic pain conditions, yielding a total of 126 associations with evidence suggestive of causality, with six metabolites implicated in more than one pain condition. Among these conditions, 63 were identified as protective factors for chronic regional pain and 63 as risk factors. Sensitivity analyses and heterogeneity tests supported the robustness of these findings. Reverse MR analysis indicated that neck and shoulder pain was associated with a decreased AMP-to–N-palmitoyl-sphingosine ratio.

**Conclusions:**

This exploratory, hypothesis-generating MR study identifies candidate plasma metabolites and metabolic pathways potentially involved in chronic pain susceptibility across multiple anatomical sites. These findings provide a foundation for future experimental and clinical studies to further investigate metabolite-related mechanisms and potential therapeutic targets.

## 1 Introduction

Chronic pain is a substantial personal and economic burden, affecting between one-third and one-half of the global population. Unlike acute pain, which serves a protective role in survival, chronic pain represents a complex neuropsychiatric condition. An incomplete understanding of its pathological mechanisms limits the development of clinically effective treatments. According to the International Association for the Study of Pain (IASP), chronic pain is defined as an undesirable sensory and emotional experience associated with actual or potential tissue damage [1]. Pain is one of the top ten reasons people seek medical attention, including headaches, back pain, and osteoarthritis [2]. Among the four leading causes of years lived with disability, three—back pain, musculoskeletal disorders, and neck pain—are chronic pain conditions [3]. Pain is generally categorized as nociceptive (resulting from tissue damage) or neuropathic (resulting from nerve damage), which has implications for assessment and decision-making.

The IASP defines chronic pain as lasting longer than three months [4]. Acute pain functions as a warning signal that protects the body from tissue damage. However, when acute pain transitions into chronic pain, it is no longer merely a symptom of injury or disease, but becomes an independent medical problem. Chronic pain significantly impairs patients’ quality of live and imposes a substantial healthcare burden on society [5,6]. There remains a limited understanding of the molecular mechanisms underlying chronic pathological pain. In addition, the safety, tolerability, and efficacy of current treatments for chronic pain in clinical practice remain inadequate [7].

In recent years, numerous studies have demonstrated associations between intestinal microorganisms and chronic physical pain conditions, including inflammatory pain [8], neuropathic pain [9], and headache [10]. With the rapid development of metabolomics, research on plasma metabolites in various diseases has expanded considerably. Cristina et al. identified serum metabolites reflecting intestinal microbiome function and demonstrated a connection between microbial community diversity and metabolic alterations related to type 2 diabetes, highlighting the important role of plasma metabolic disorders [11]. Several studies have also suggested links between circulating metabolites and chronic pain. Linsong et al. conducted an MR analysis exploring the relationship between 249 plasma metabolites and back pain, identifying four metabolites-lactate, medium low–density lipoprotein triglycerides, albumin, and tyrosine – as potentially causally involved in low back pain (LBP) [12]. Silva and colleagues examined the association between hypovitaminosis D and low back pain in 9305 postmenopausal women and found that individuals with hypovitaminosis D had a higher risk of back pain [13]. Shan et al. reported that higher plasma omega-3 concentrations were associated with a reduced risk of LBP using MR analysis, suggesting that omega-3 fatty acid supplementation may play a role in prevention and treatment [14]. Gary et al. demonstrated through metabolomics analysis that sphingomyelin-ceramide metabolism was altered in the dorsal horn of rats with neuropathic pain, suggesting that abnormalities in this pathway may contribute to neuropathic pain and represent potential therapeutic targets [15]. Collectively, these findings highlight the potential value of metabolomics in chronic pain research, including improving mechanistic understanding, enabling risk prediction, and supporting the development of personalized approaches to pain managemen.

Despite these advances, robust plasma metabolite markers for predicting site-specific chronic pain remain limited, partly due to constraints in sample size and metabolomic coverage in existing studies. To further investigate the metabolic mechanisms underlying chronic pain and identify potential target for prevention and intervention, we selected eight regional pains phenotypes and conducted an MR study to systematically explore the causal relationship between blood metabolites and site-specific chronic pain.

Metabolites are intermediates or end products of metabolic processes. Because metabolite levels reflect the activity of numerous metabolic pathways, detecting plasma metabolites may improve disease understanding, diagnosis, and management. To date, however, the application of metabolomics in pain medicine remains relatively limited. Establishing causal links between metabolites and disease etiology may more reliable evidence for precision medicine intervention [16]. MR is a causal inference method that uses genetic variation as instrumental variable (IV) to evaluate the effect of an exposure on an outcome. Compared with randomized controlled trials (RCTs), MR is less susceptible to confounding and reverse causation, thereby reducing bias in causal estimation [[17], [18], [19]].

In this context, we conducted an exploratory, hypothesis-generating two-sample MR analysis using GWAS summary statistics for 1400 plasma metabolites and eight site-specific chronic pain phenotypes. The primary aim was not to establish definitive causal mechanisms, but to identify and prioritize candidate metabolite–pain relationships and metabolic pathways that may warrant further experimental and clinical investigation.

## 2 Methods

### 2.1 Study design

This study was designed as an exploratory, hypothesis-generating MR analysis rather than a confirmatory causal investigation.

### 2.2 GWAS data for exposure—plasma metabolites

GWAS data on plasma metabolites from Brent Richards' research included 1091 metabolites and 309 metabolite ratios measured in 8299 individuals from the Canadian Longitudinal Study of Aging (CLSA) cohort [20]. Among the 1091 plasma metabolites teste, 850 are known to participate in eight super-pathways, such as lipids, amino acids, and nucleic acids, while the remaining 241 are small molecules that have not been fully characterized. The study also includes 81 metabolites that have not been included in previous large-scale GWASs.

Among the 309 metabolic ratios, 69 loci were identified, of which 63 metabolic ratio-locus associations were newly discovered. The authors further identified 16 genome-wide significant genetic variants, suggesting that metabolic ratios may help identify novel genetic determinants. Super-pathways analysis indicated that most associated metabolites were involved in amino acids, lipids, and energy-related pathways. Complete GWAS summary statistics for all 1091 plasma metabolites and 309 metabolites ratio are publicly available in NHGRI-EBI GWAS Catalog (https://www.ebi.ac.uk/gwas/).

### 2.3 GWAS data for outcome—chronic pain

Data for chronic regional pain (CRP) were obtained from the IEU open GWAS project (v7.5.5-2023-08-09, n = 42,348), derived from the UK Biobank cohort. In the UK Biobank, chronic pain is defined using a temporal criterion of pain persisting for more than three months. Accordingly, pain lasting longer than three months was selected and categorized into eight site-specific phenotypes: headache, facial pain, neck or shoulder pain, stomachache, back pain, knee pain, general pain, and hip pain. All participants in this study were of European ancestry. Full details of CRP phenotypes are provided in Supplementary Table S1.

### 2.4 Selection of genetic instruments

Single nucleotide polymorphisms (SNPs) associated with human plasma metabolome prioritizes were obtained from the GWAS dataset reported by Chen et al. [20]. Instrumental variables (IVs) were selected as SNPs significantly associated with in published GWAS studies. The screening criteria included SNPs independently associated with plasma metabolites at *p* < 1 × 10^−5^ with an F-statistic >10 to minimize weak instrument bias.

We then performed linkage disequilibrium (LD) clumping using a European reference panel from the 1000 Genomes project to select independent SNPs with (*r*^2^ < 0.001 within the 10,000 kb window). The F-statistic was used to assess instrument strength of each metabolite. Instruments with F < 10 were considered weak and were removed.

### 2.5 Mendelian randomization analyses

**Fig. 1:**
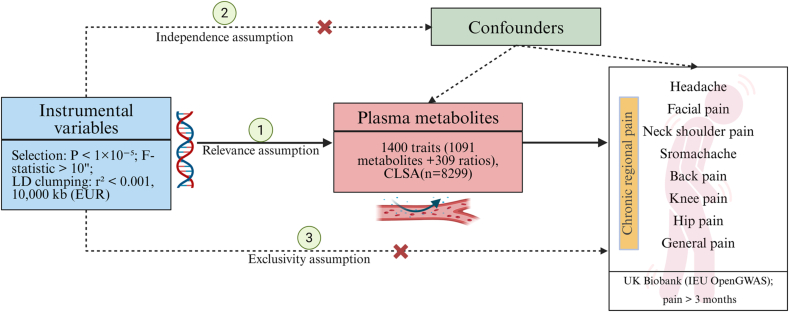
Diagram of the MR three assumptions for investigating the causal association of plasma metabolites and chronic pain.

MR analysis is based on three core assumptions: (1) Relevance assumption: The selected genetic variants (IVs) must be significantly associated with the exposure of interest; (2) Exclusion restriction assumption: The IVs influence the outcome only through the exposure and not through alternative pathways; (3) Independence assumption: The IVs are not associated with confounding factors affecting expose-outcome relationship (Fig. 1). After satisfying these assumptions, causality inference between exposure and outcome can be evaluated.

To assess the potential causal relationship between plasma metabolites and site-specific chronic pain risks, we performed a two-sample MR analysis in which the genetic association with exposure and outcome were derived from two independent, non-overlapping datasets. Five MR methods were applied: inverse variance weighted (IVW), MR Egger, Weighted mode, Simple mode, and Weighted median. The IVW method was considered the primary analysis.

**Fig. 2:**
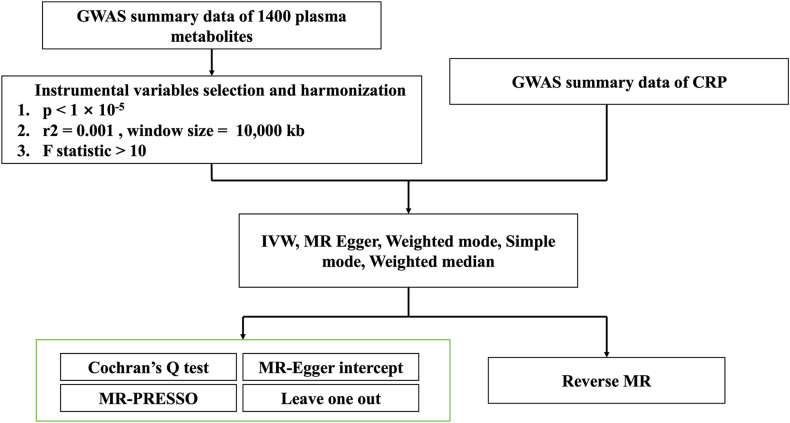
MR schematic and workflow. GWAS, genome-wide association study; CRP, chronic pain; IVW, inverse variance weighted; SNPs, single nucleotide polymorphisms; kb, kilobase; MR-PRESSO, Mendelian Randomization Pleiotropy RESidual Sum and Outlier.

In IVW results, an odds ratio (OR) < 1 was interpreted as a protective association, whereas an OR>1 indicated a risk association. To ensure the robustness of our findings, a causal association was identified as a positive result only if the IVW method showed nominal significance (*P* < 0.05) and the direction of effect estimates was entirely consistent across all five methods. Reverse MR analyses were also conducted to examine the potential reverse causal relationship between plasma metabolites and chronic pain phenotypes (Fig. 2).

### 2.6 Heterogeneity and sensitivity analysis

Cochran's Q test was used to assess heterogeneity, with *p* < 0.05 indicating significant. The MR-Egger intercept test and MR-PRESSO were applied to detect horizontal pleiotropy; a p-value ≥0.05 indicated no significant pleiotropy. Leave-one-out analysis was performed to evaluate the robustness of the results (Fig. 2). All analyses were performed using R packages “Two Sample MR” and “MR-PRESSO” in R software (version 4.2.3). Schematics were created with BioRender.com.

## 3 Results

The purpose of this study was to investigate the possible causal relationship between 1400 plasma metabolites and eight types of chronic regional pain. We extracted IVs associated with plasma metabolites from the previous research [20], and 112 SNPs associated with chronic pain were selected as potential IVs at a significance threshold of *p* < 1 × 10^−5^. Detailed information on these SNPs, including the effect allele, beta coefficient, standard error, and *p*-value, is provided in Supplementary materials 1.

**Fig. 3:**
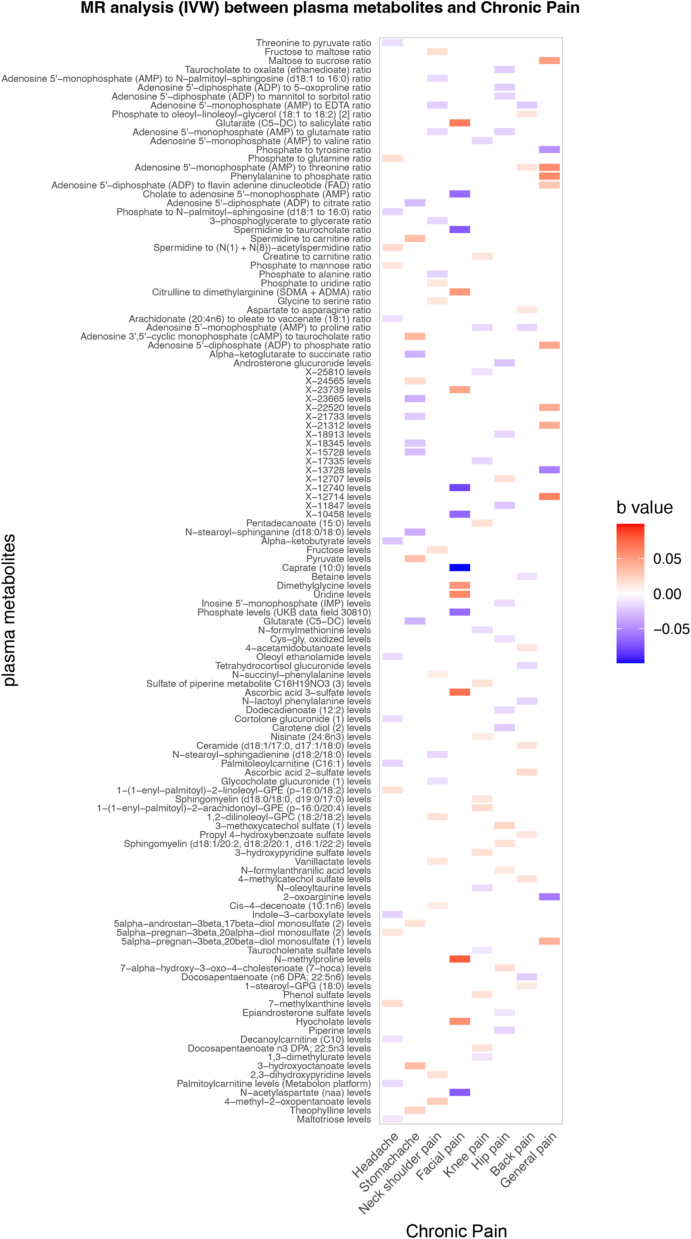
Comprehensive summary heatmap showing the associations between plasma metabolites and different chronic pain phenotypes.

After instrument filtering (F-statistic >10), 122 plasma metabolites were retained for MR analysis across eight chronic pain phenotypes. Across outcomes, we identified genetically predicted associations between metabolites and chronic headache (17), facial pain (15), neck-shoulder pain (17), back pain (15), abdominal pain (15), knee pain (17), general pain (12), and hip pain (18). Effect sizes were generally modest and varied in direction across phenotypes (Fig. 3).

### 3.1 Causal effects of plasma metabolites on different chronic pain

#### 3.1.1 Headache

**Supplementary Fig. 1:**
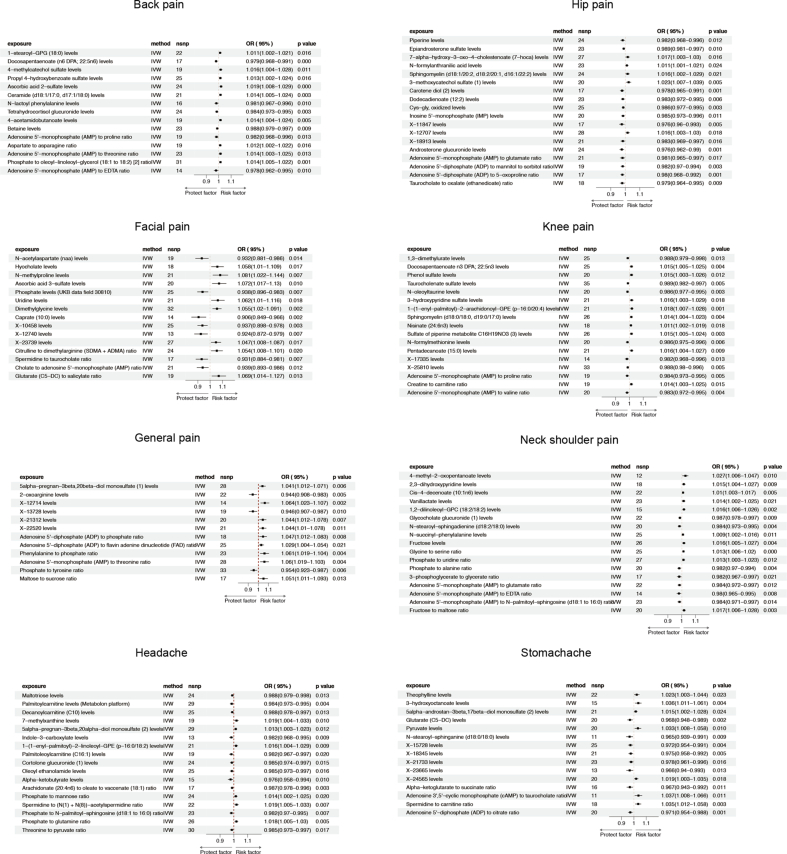
**Forest plot of presumptive causal relationships between plasma metabolites and chronic pain.** The vertical (dashed) line in the middle is a null line with OR = 1, indicating no statistical difference between exposure and outcome. The small square and the horizontal bars indicate the estimated OR and the 95% CI, respectively.

We initially identified 64 metabolites with possible causal associations with chronic headache. After sensitivity analysis, including leave-one-out testing, 17 metabolites remained. IVW analysis showed significant correlations between these plasma metabolites and chronic headaches (Fig. S1; Supplementary Table S2).

Among the 17 metabolites, 11 were associated with a reduced risk of chronic headache. The most notable were histidine (OR: 0.981, 95%CI: 0.968-0.995, *p* = 0.007), indole-3-carboxylate levels (OR: 0.982, 95%CI: 0.968−0.995, *p* = 0.009), phosphate to N-palmitoyl-sphingosine (d18:1 to 16:0) ratio (OR: 0.982, 95%CI: 0.97-0.995, *p* = 0.007), palmitoleoylcarnitine (C16:1) levels (OR: 0.982, 95%CI: 0.967-0.997, *p* = 0.020) and choline to taurocholate ratio (OR: 0.983, 95%CI: 0.97-0.996, *p* = 0.011) (Fig. S1 and Supplementary Table S2).

Conversely, six metabolites were associated with an increased risk of chronic headache. The strongest associations were observed for 7−methylxanthine (OR:1.019, 95%CI: 1.004-1.033, *p* = 0.010) and the spermidine to (N (1) + N (8))-acetylspermidine ratio (OR:1.019, 95%CI: 1.005-1.033, *p* = 0.007), followed by phosphate to glutamine ratio (OR:1.018, 95%CI: 1.005-1.03, *p* = 0.005), 1-(1-enyl-palmitoyl)-2-linoleoyl-GPE (p-16:0/18:2) levels (OR:1.016, 95%CI: 1.004-1.029, *p* = 0.009) and Glutamine conjugate of C7H12O2 levels (OR:1.016, 95%CI: 1.004-1.029, *p* = 0.011) (Fig. S1 and Supplementary Table S2).

Reverse MR analysis suggested a potential association between chronic pain and increased 7-methylxanthine levels in IVW analysis; however, this finding did not remain robust in the leave-one-out test (Supplementary Table S2).

#### 3.1.2 Facial pain

Using IVW analysis, 62 plasma metabolites were initially associated with chronic facial pain. After sensitivity analysis, including the leave-one-out test, 15 metabolites remained. Among these, 7 were negatively associated and 8 were positively associated with the outcome.

Among the negatively associated metabolites, the strongest association was observed for Caprate (10:0) levels (OR: 0.906, 95%CI: 0.849-0.966, *p* = 0.002), followed by X-12740 levels (OR:0.924, 95%CI: 0.872-0.979, *p* = 0.007), spermidine to taurocholate ratio (OR: 0.931, 95%CI: 0.884-0.981, *p* = 0.007) and N-acetyl aspartate (naa) levels (OR:0.932, 95%CI:0.881-0.986, *p* = 0.014) (Fig. S1 and Supplementary Table S3).

Among the positively associated metabolites, the strongest association was observed for N-methylproline levels (OR:1.081, 95%CI:1.022-1.144, *p* = 0.007), followed by ascorbic acid 3-sulfate levels (OR:1.072, 95%CI: 1.017-1.13, *p* = 0.010), glutarate (C5-DC)- to-salicylate ratio (OR:1.069, 95%CI: 1.014-1.127, *p* = 0.013), and uridine levels (OR:1.062, 95%CI: 1.01-1.116, *p* = 0.018). (Fig. S1 and Supplementary Table S3).

#### 3.1.3 Neck shoulder pain

Based on the IVW analysis, a total of 75 metabolite were initially identified as potentially associated with chronic neck-shoulder pain. After heterogeneity tests and sensitivity analysis, 17 metabolites remained, of which 10 showed positive associations and 7 showed negative associations with neck-shoulder pain.

Among the negatively associated metabolites were the adenosine 5′-monophosphate (AMP) to EDTA ratio (OR: 0.980, 95%CI: 0.965-0.995, *p* = 0.008), phosphate to alanine ratio (OR: 0.982, 95%CI: 0.97-0.994, *p* = 0.004), 3-phosphoglycerate to glycerate ratio (OR: 0.982, 95%CI: 0.967-0.997, *p* = 0.021), N-stearoyl-sphingadienine (d18:2/18:0) levels (OR: 0.984, 95%CI: 0.973-0.995, *p* = 0.004), Adenosine 5′-monophosphate (AMP) to glutamate ratio (OR: 0.984, 95%CI: 0.972-0.997, *p* = 0.012), and Adenosine 5′-monophosphate (AMP) to N-palmitoyl-sphingosine (d18:1 to 16:0) ratio (OR: 0.984, 95%CI: 0.971-0.997, *p* = 0.008).

Among the positively associated metabolites, the strongest association was observed for 4-methyl-2-oxo pentanoate levels (OR: 1.027, 95%CI: 1.006-1.047, *p* = 0.010), followed by the fructose to maltose ratio (OR: 1.017, 95%CI: 1.006-1.028, *p* = 0.003), 1,2-dilinoleoyl-GPC (18:2/18:2) levels (OR: 1.016, 95%CI: 1.006-1.026, *p* = 0.002), and fructose levels (OR: 1.016, 95%CI: 1.005-1.027, *p* = 0.004) (Fig. S1 and Supplementary Table S4).

Reverse MR analysis suggested a potential association between chronic neck–shoulder pain and Adenosine 5′-monophosphate (AMP) to N-palmitoyl-sphingosine (d18:1 to 16:0) ratio. However, two metabolites-the phosphate to alanine ratio, and AMP-to-glutamate ratio-were excluded due to inconsistency in the leave-one-out analysis.

#### 3.1.4 Back pain

IVW analysis initially identified 86 plasma metabolites associated with chronic back pain. After sensitivity analysis and leave-one-out analysis, 15 metabolites remained, including 6 were negatively associated metabolites. Among the negatively associated metabolites, the AMP-to-EDTA ratio (OR: 0.978, 95%CI: 0.962-0.995, *p* = 0.010) showed the strongest, followed by docosapentaenoate (n6 DPA; 22:5n6) levels (OR:0.979, 95%CI: 0.968-0.991, *p* = 0.000).

Conversely, the most significant positive association was observed for ascorbic acid 2-sulfate levels (OR: 1.019, 95%CI: 1.008-1.029, *p* = 0.000), followed by 4-methylcatechol sulfate levels (OR: 1.016, 95%CI: 1.004-1.028, *p* = 0.011), ceramide (d18:1/17:0, d17:1/18:0) ratio (OR: 1.014, 95%CI: 1.005-1.024, *p* = 0.003), 4-acetamidobutanoate levels (OR: 1.014, 95%CI: 1.004-1.024, *p* = 0.005), AMP-to- threonine ratio (OR: 1.014, 95%CI: 1.003-1.025, *p* = 0.013), and phosphate to oleoyl-linoleoyl-glycerol (18:1 to 18:2) ratio (OR: 1.014, 95%CI: 1.005-1.022, *p* = 0.001) (Fig. S1 and Supplementary Table S5).

#### 3.1.5 Stomachache

IVW analysis initially identified 66 plasma metabolites associated with chronic stomachache. After sensitivity analysis, 15 metabolites remained, including 8 metabolites negatively and 7 metabolites positively associated metabolites.

Among the negatively associated metabolites were N-stearoyl-sphinganine (d18:0/18:0) levels (OR:0.9965, 95%CI: 0.939-0.991, *p* = 0.009), X-23665 (OR:0.966, 95%CI: 0.94-0.993, *p* = 0.013), phenylacetylcarnitine (OR:0.967, 95%CI: 0.948-0.986, *p* = 0.001), and glutarate (C5-DC) (OR:0.968, 95%CI: 0.948-0.989, *p* = 0.002), suggesting potential protective associations.

Among the positively associated metabolites were the Adenosine 3′,5′-cyclic monophosphate (cAMP) to taurocholate ratio (OR: 1.037, 95%CI: 1.008-1.066, *p* = 0.011), 3-hydroxyoctanoate levels (OR: 1.036, 95%CI: 1.011-1.061, *p* = 0.004), AMP levels (OR: 1.036, 95%CI: 1.006-1.067, *p* = 0.017), and Spermidine to carnitine ratio (OR: 1.035, 95%CI: 1.012-1.058, *p* = 0.003), indicating that higher levels may be associated with increased risk (Fig. S1 and Supplementary Table S6).

#### 3.1.6 Knee pain

IVW analysis identified 63 plasma metabolites associated with chronic knee pain. After sensitivity analysis and leave-one-out analysis, 17 metabolites remained. Among these, 8 were negatively associated and nine were positively associated with knee pain.

Among the negatively associated metabolites, X-17335 levels (OR: 0.982, 95%CI: 0.968-0.996, *p* = 0.013) showed the strongest association, followed by AMP-to-valine ratio (OR: 0.983, 95%CI: 0.972-0.995, *p* = 0.004), and AMP-to-proline ratio (OR: 0.984, 95%CI: 0.973-0.995, *p* = 0.005).

Among the positively associated metabolites, 1-(1-enyl-palmitoyl)-2-arachidonoyl-GPE (p-16:0/20:4) levels (OR: 1.018, 95%CI: 1.007-1.028, *p* = 0.001) showed the strongest association, followed by 3-hydroxypyridine sulfate (OR: 1.016, 95%CI: 1.003-1.029, *p* = 0.018), pentadecanoate (15:0) levels (OR: 1.016, 95%CI: 1.004-1.027, *p* = 0.009), docosapentaenoate n3 DPA; 22:5n3 levels (OR: 1.015, 95%CI: 1.005-1.025, *p* = 0.004), phenol sulfate levels (OR:1.015, 95%CI: 1.003-1.026, *p* = 0.012), and sulfate of piperine metabolite C16H19NO3 (3) levels (OR: 1.015, 95%CI: 1.005-1.024, *p* = 0.003), suggesting potential risk associations (Fig. S1 and Supplementary Table S7).

#### 3.1.7 General pain

We identified 12 metabolites associated with chronic general pain. Among these, nine showed positive associations and three showed negative associations.

Among the positively associated metabolites, X-12714 levels (OR: 1.064, 95% CI: 1.023-1.107, *p* = 0.002) showed the strongest association, followed by Phenylalanine to phosphate ratio (OR: 1.061, 95% CI: 1.019-1.104, *p* = 0.004), AMP-to-threonine ratio (OR: 1.06, 95% CI: 1.019-1.103, *p* = 0.004), suggesting potential risk associations.

Among the negatively associated metabolites, the phosphate to tyrosine ratio (OR: 0.954, 95% CI: 0.923-0.987, *p* = 0.006) showed the strongest association, followed by 1,3-dimethylurate levels (OR: 0.953, 95% CI: 0.918-0.991, *p* = 0.015), 2-oxoarginine levels (OR: 0.944, 95% CI: 0.908-0.983, *p* = 0.005), and X-13728 levels (OR: 0.946, 95% CI: 0.907-0.987, *p* = 0.010), suggesting potential protective associations (Fig. S1 and Supplementary Table S8).

#### 3.1.8 Hip pain

For the hip pain part, IVW analysis initially identified 55 plasma proteins associated with chronic hip pain. After sensitivity analysis and leave-one-out analysis, 18 metabolites remained, including 13 negatively and 5 positively associated metabolites.

Among the negatively associated metabolites, X-11847 levels (OR: 0.976, 95%CI: 0.96-0.993, *p* = 0.005) and Androsterone glucuronide levels (OR: 0.976, 95%CI: 0.962-0.990, *p* = 0.001) showed the strongest association, followed by carotene diol levels (OR: 0.978, 95%CI: 0.965-0.991, *p* = 0.001), and taurocholate to oxalate (ethanedioate) ratio (OR: 0.979, 95%CI: 0.964-0.995, *p* = 0.009).

Among the positively associated metabolites were 3-methoxy catechol sulfate (1) levels (OR: 1.023, 95%CI: 1.007-1.039, *p* = 0.005), 7-alpha-hydroxy-3-oxo-4-cholestenoate (7-hoca) levels (OR: 1.017, 95%CI: 1.003-1.03, *p* = 0.016), Sphingomyelin (d18:1/20:2, d18:2/20:1, d16:1/22:2) levels (OR: 1.016, 95%CI: 1.002-1.029, *p* = 0.021), and X-12707 levels (OR: 1.016, 95%CI: 1.003-1.03, *p* = 0.018) (Fig. S1 and Supplementary Table S9).

#### 3.1.9 Sensitivity and reverse MR analyses

MR-Egger regression and MR-PRESSO test suggested no evidence of horizontal pleiotropy between exposure and outcomes. Cochran's Q-test also indicated no significant heterogeneity across chronic pain phenotypes. Leave-one-out analysis further supported the robustness of the findings (Supplementary Table S10).

In reverse MR analysis, only neck-shoulder pain showed a potential association with plasma metabolites (Supplementary Table S10). Specifically, the AMP to N-palmitoyl-sphingosine (d18:1 to 16:0) ratio (OR: 3.069, 95% CI: 1.332-7.068, *p* = 0.008) was potentially associated with neck-shoulder pain. No significant associations were observed in IVW analysis for back pain, general pain, knee pain, and stomachache.

Although facial pain, headache, and hip pain showed nominal associations in IVW analysis, these were excluded due to inconsistency in pleiotropy analysis and leave-one-out analysis (Supplementary Table S12–13).

Given the exploratory nature of the study and modest effect sizes observed, these findings should be interpreted as identifying candidate metabolic pathways rather than establishing definitive causal effects.

## 4 Discussion

**Fig. 4:**
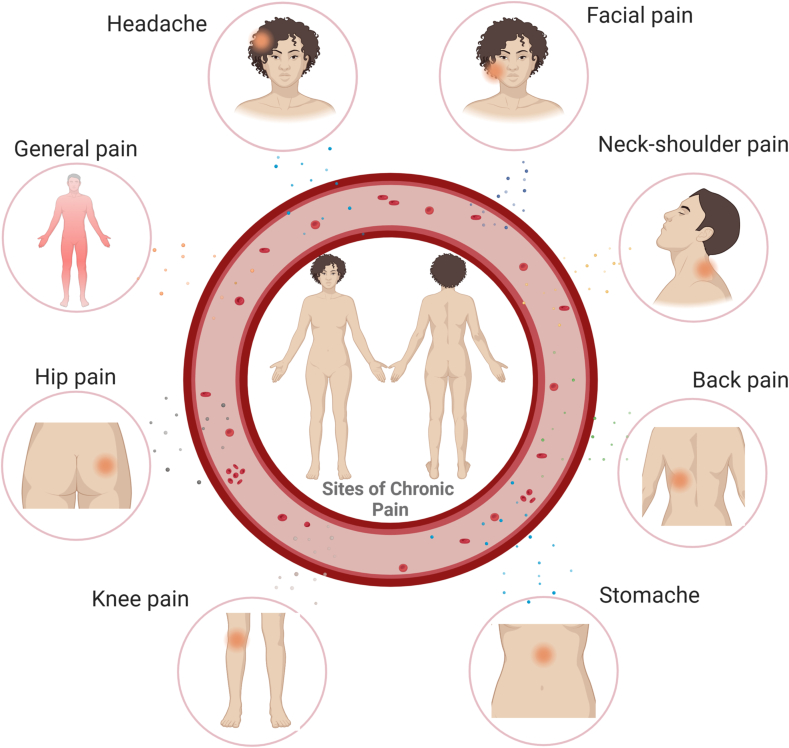
Schematic illustration of the causal relationship between plasma metabolites and chronic pain.

**Supplementary Fig. 2:**
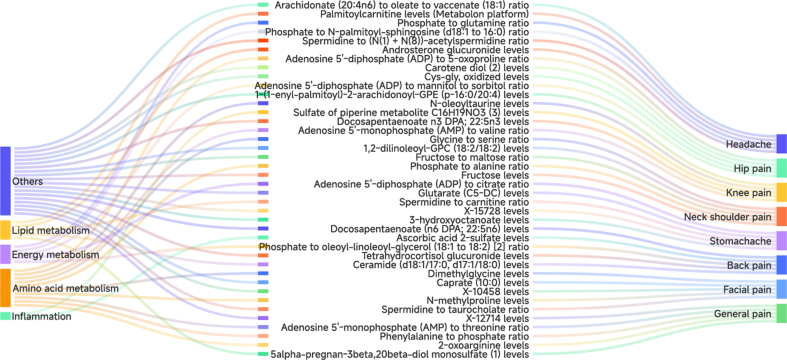
Causal network of plasma metabolites and chronic regional pain.

Based on the existing literature, this study provides an exploratory framework for investigating potential causal relationships between plasma metabolites and site-specific chronic pain phenotypes. Using a two-sample MR analysis, we identified genetically predicted associations between plasma metabolites and eight chronic pain sites, including headache, facial pain, neck-shoulder pain, back pain, knee pain, general pain, and hip pain (Fig. 4), and constructed a causal network linking plasma metabolites with chronic regional pain (Supplementary Fig. 2). After sensitivity analyses, 122 metabolites showed potential causal links across pain phenotypes, with varying directions and magnitudes of association. Rather than indicating definitive causal effects, these findings nominate biologically plausible metabolic pathways that may contribute to chronic pain susceptibility and warrant further mechanistic investigation.

Pain originates from nociceptors in peripheral tissues that transform noxious stimuli into neural signals transmitted to the spinal dorsal horn and subsequently to supraspinal regions such as the thalamus, somatosensory cortex, and anterior cingulate cortex via the spinothalamic tract [1,21,22]. Increasing evidence indicates that non-neuronal cells, such as glial cells, immune cells, and even tumor cells, are also involved in pain regulation throughout the peripheral and central nervous systems [23,24]. A better understanding of systemic metabolic and immune regulation of these pathways may therefore improve the management of chronic pain.

### 4.1 Pathway-level interpretation of metabolite associations

Rather than focusing solely on individual metabolites, our findings highlight clustering within several biologically plausible metabolic pathways. Many identified associations were enriched in pathways related to amino acid metabolism, purine and energy metabolism, lipid signaling, and inflammatory mediators. These pathways are closely linked to mitochondrial function, neuroimmune signaling, and central sensitization, all of which are increasingly recognized as key contributors to chronic pain pathophysiology.

Growing evidence supports the role of systemic metabolic and inflammatory changes in chronic pain conditions. For example, studies of Complex regional pain syndrome (CRPS) have demonstrated elevated levels of proinflammatory neuropeptides, cytokines (IL-1β, IL-2, and IL-6), and calcitonin gene-related peptides are elevated in the plasma and cerebrospinal fluid during early disease stages [[25], [26], [27]]. Passive transfer of serum IgG from CRPS patients into mice induces persistent hyperalgesia, which can be reversed by interleukin-1 blockade, highlighting the importance of circulating factors in pain modulation [28]. Metabolomic analyses in musculoskeletal pain conditions have also revealed shifts in plasma metabolic profiles following treatment, including changes in amino acids, carnitine, and bilirubin [29]. Similarly, postoperative pain improvement after knee arthroplasty has been associated with reductions in substance P and increases in anti-inflammatory cytokines [30]. However, many of these studies remain observational and are limited by confounding and reverse causality. Genetic approaches such as MR can help prioritize candidate metabolic pathways for further mechanistic investigation. Indeed, GWAS evidence suggests that chronic pain risk is polygenic and involves numerous weak but interacting genetic effects [31]. Therefore, a comprehensive MR analysis exploring the relationship between plasma metabolites and chronic pain is warranted.

### 4.2 Amino acid metabolism and excitatory neurotransmission

Amino acid metabolism emerged as one of the most prominent pathway-level signals in our analysis. Numerous studies have demonstrated that glutamate, the principal excitatory neurotransmitter, plays an important role in primary sensory neurotransmission and pain and central sensitization across multiple pain conditions, including headaches, trigeminal neuralgia, and chronic widespread pain [[32], [33], [34]]. Elevated plasma glutamate and aspartate levels have been reported during migraine attacks, whereas histidine levels tend to be reduced [35] and glutamate levels correlate with attack duration [36]. In line with these findings, we observed that the Adenosine 5′-monophosphate (AMP)-to-glutamate ratio was inversely associated with neck–shoulder and hip pain risk, suggesting that relative changes in energy metabolism and excitatory neurotransmission may influence regional pain susceptibility. Alterations in glutamate, ATP, pyruvate, and lactate have also been reported in chronic widespread pain, supporting a broader role for metabolic dysregulation in pain modulation [[37], [38], [39]].

### 4.3 Energy metabolism and purinergic signaling

Beyond individual metabolites, the results point to potential involvement of energy and purine metabolism pathways, particularly those related to AMP and adenosine signaling. AMP participates in ATP synthesis and cellular energy metabolism, and the AMP/ATP ratio regulates AMPK activity. In recent years, Hong et al. found that the AMPK signaling pathway can alleviate inflammatory pain by inhibiting NF-kB activation and IL-1β expression [40]. AMPK activation can also attenuate inflammatory pain by promoting macrophage polarization [41]. These findings may help explain why the AMP/glutamate ratio appeared protective for neck-shoulder pain and hip pain in our study.

In addition, consistent with previous studies [34,35], histidine levels were notably negatively associated with headache risk in our study. The aspartate to asparagine ratio was positively associated with back pain, suggesting that increased aspartate levels may elevate back pain risk.

### 4.4 Lipid and steroid metabolism

We also identified signals related to steroid metabolism and lipid-related pathways, such as epiandrosterone sulfate (EAS), which was negatively associated with the risk of hip pain, and has previously been associated with chronic widespread musculoskeletal pain [42]. Such metabolites may influence pain through interactions with inflammatory signaling, neuroendocrine regulation, and membrane lipid composition affecting neuronal excitability. Collectively, these observations support the concept that chronic pain is linked to systemic metabolic–immune interactions rather than isolated molecular changes.

Fais et al. evaluated serum purine levels in patients with fibromyalgia (FM) and found that inosine, hypoxanthine, and xanthine levels were significantly increased, whereas adenine levels were significantly decreased [43]. These discrepancies may reflect differences in pain phenotype, tissue specificity, or metabolic context. Taken together, these observations support the concept that chronic pain reflects systemic metabolic–immune interactions rather than isolated molecular changes.

### 4.5 Site-specific versus shared metabolic mechanisms

An important observation from our analysis is the heterogeneity across pain sites. While some metabolic pathways—particularly those related to amino acid and energy metabolism—appear to represent shared mechanisms, others may be region-specific. Differences in tissue innervation, biomechanical load, immune microenvironment, and central processing may all influence how systemic metabolic alterations translate into localized pain phenotypes.

For example, several AMP-related ratios showed protective associations in neck-shoulder and back pain but risk associations in general pain. Adenosine, derived from AMP, exerts antinociceptive effects via A1 receptor activation in nociceptive neurons and spinal cord pathways [44,45]. Intrathecal adenosine administration has been reported to reduce hyperalgesia in chronic pain models and clinical settings [46,47]. In our analysis, several AMP-related metabolite ratios showed associations with different pain phenotypes, suggesting that energy metabolism and purinergic signaling may represent shared mechanistic pathways across pain sites, although the direction and magnitude of associations varied by phenotype.

### 4.6 Strengths and limitations

This study has several limitations. First, to obtain an adequate number of instrumental variables, we used a relatively relaxed SNP selection threshold, which may introduce weaker genetic instruments despite applying F-statistic screening. Second, the GWAS data were restricted to individuals of European ancestry, limiting generalizability to other populations. Third, chronic pain phenotypes in the UK Biobank are based on self-reported pain lasting more than three months and lack detailed clinical characterization such as severity, etiology, and treatment history, which may contribute to phenotype heterogeneity. Fourth, although MR can strengthen causal inference, the modest effect sizes and multiple testing burden require cautious interpretation. Finally, lack of individual-level data prevented more detailed stratified analyses.

Despite these limitations, this study provides a systematic and hypothesis-generating overview of potential metabolite–pain relationships across multiple anatomical sites. By highlighting metabolic pathways—particularly those related to amino acid metabolism, energy metabolism, and inflammatory signaling—our findings help prioritize candidate mechanisms for future experimental and clinical research.

## 5 Conclusion

In summary, this exploratory Mendelian randomization study systematically evaluated genetic evidence linking circulating plasma metabolites to multiple site-specific chronic pain phenotypes. The findings highlight several metabolite classes and metabolic pathways—including amino acid metabolism, energy metabolism, and lipid-inflammatory signaling—that may be involved in chronic pain susceptibility. However, these associations should be interpreted cautiously and considered hypothesis-generating. Further experimental and clinical studies are needed to clarify underlying mechanisms and potential therapeutic relevance.

## CRediT authorship contribution statement

**Yanwen Li:** Supervision, Data curation. **Muzi Li:** Software, Project administration, Methodology, Formal analysis. **Kang Peng:** Software, Formal analysis, Data curation. **Wei Zhang:** Software, Project administration, Methodology, Data curation. **Liling Guo:** Writing – review & editing, Writing – original draft, Supervision, Software, Data curation. **Long Chen:** Supervision, Funding acquisition.

## Ethics approval

Not applicable.

## Funding

This work was supported by “10.13039/501100009076USTC Research Funds of the Double First-Class Initiative” (YD9110002120) and “Natural Science Foundation of Anhui Province” (2508085QH342).

## Competing interests

The authors have no relevant financial or non-financial interests to disclose.

## Data Availability

The datasets used and analyzed during the current study are available from the corresponding author upon reasonable request.
