# Supplementary figures and images for: Causal relationship between plasma metabolites and chronic regional pain: a Mendelian randomization study
Source: Metabol Open. 2026 Mar 7;30:100456. doi: 10.1016/j.metop.2026.100456 (PMC12996675; doi:10.1016/j.metop.2026.100456)

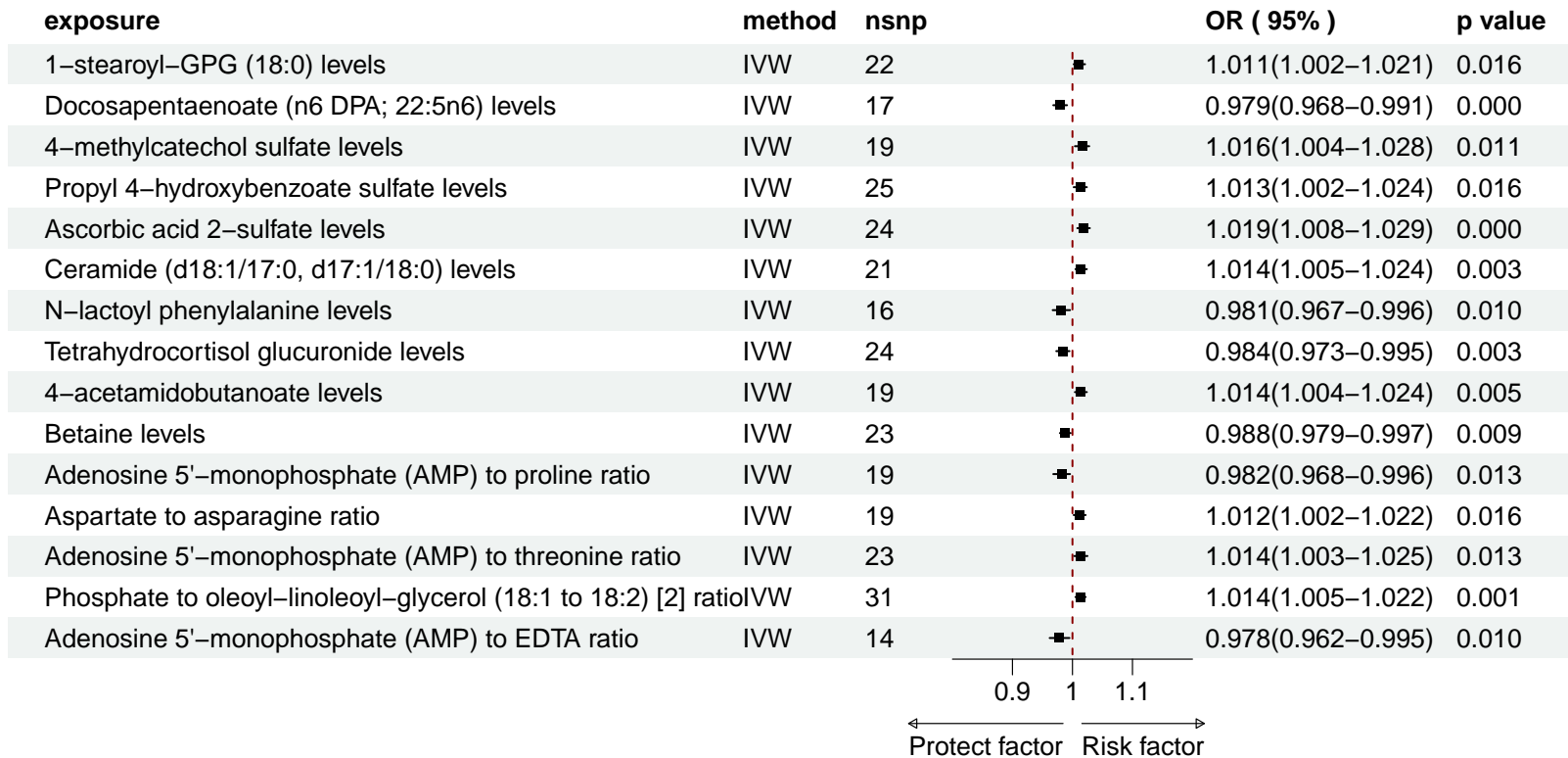

Supplement: Multimedia component 1 [file mmc1.pdf]

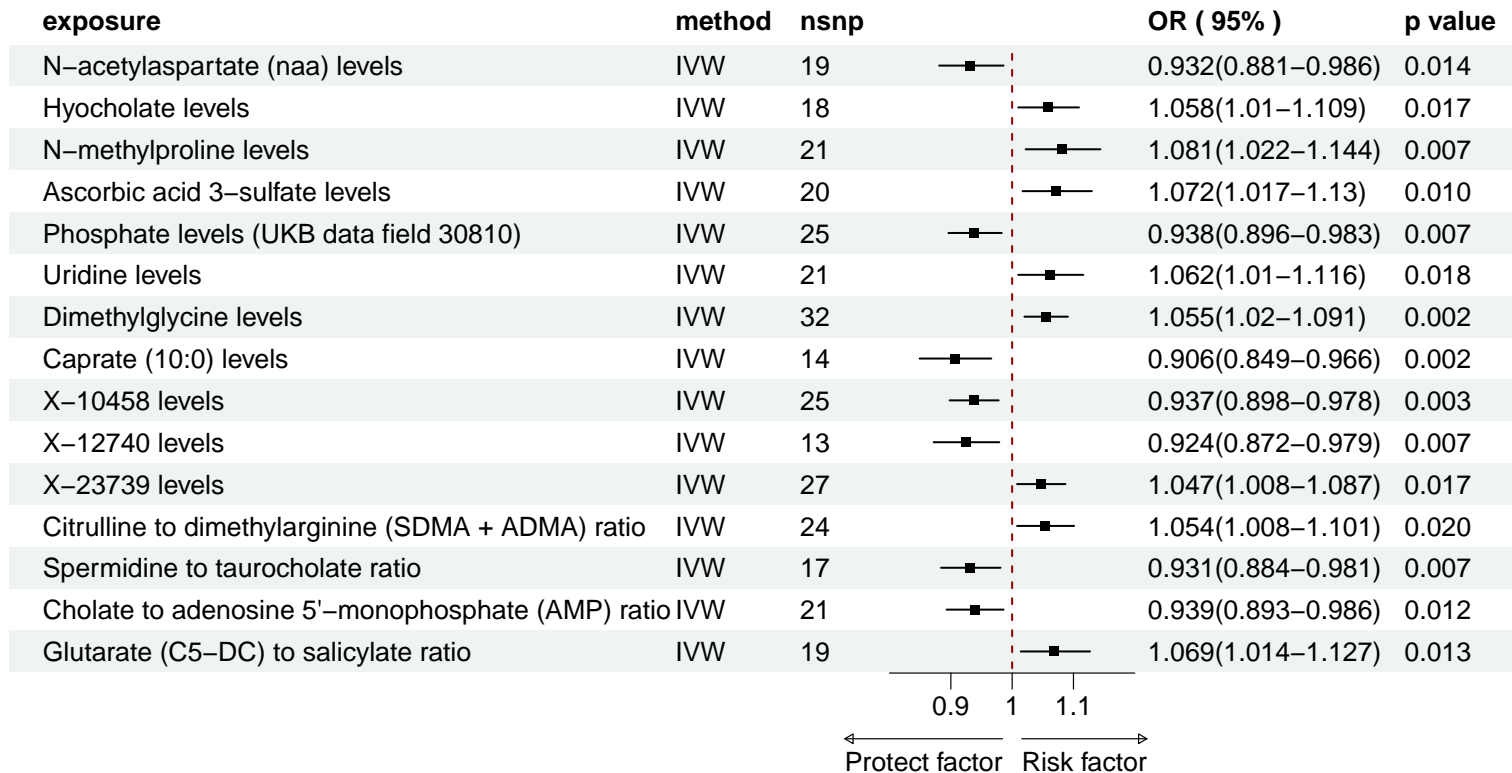

Supplement: Multimedia component 2 [file mmc2.pdf]

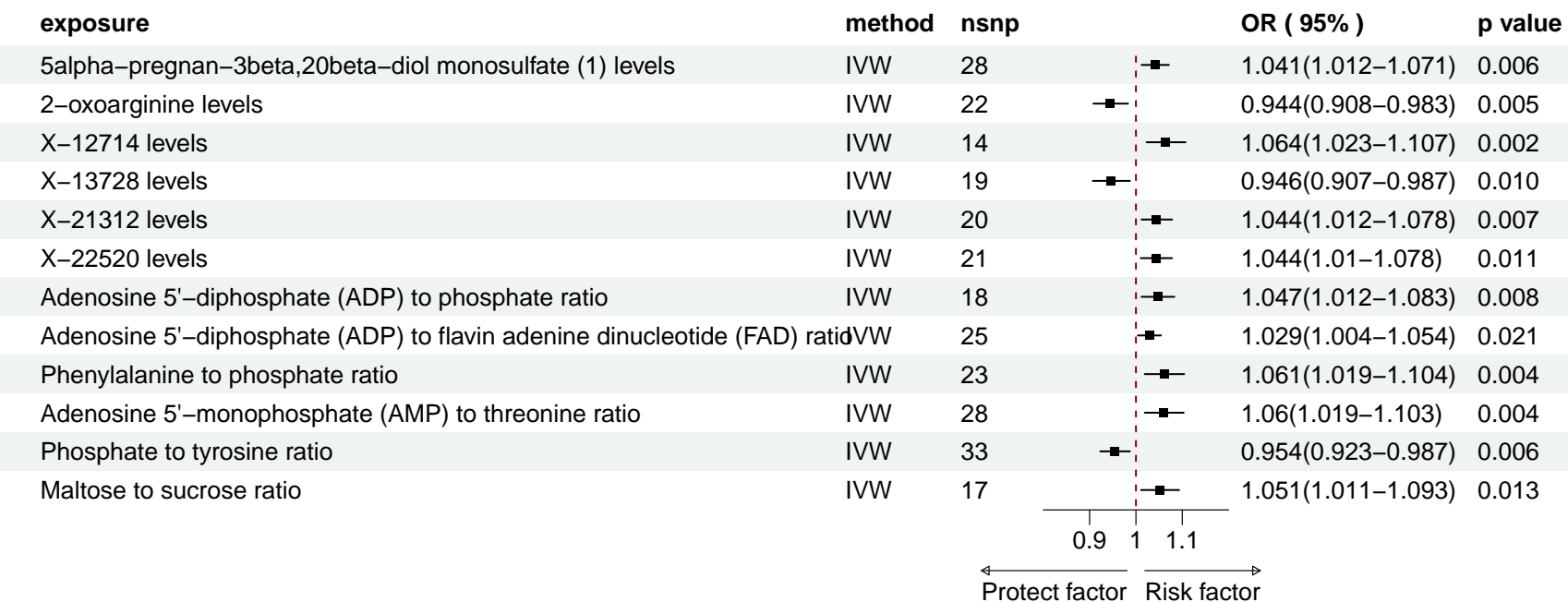

Supplement: Multimedia component 3 [file mmc3.pdf]

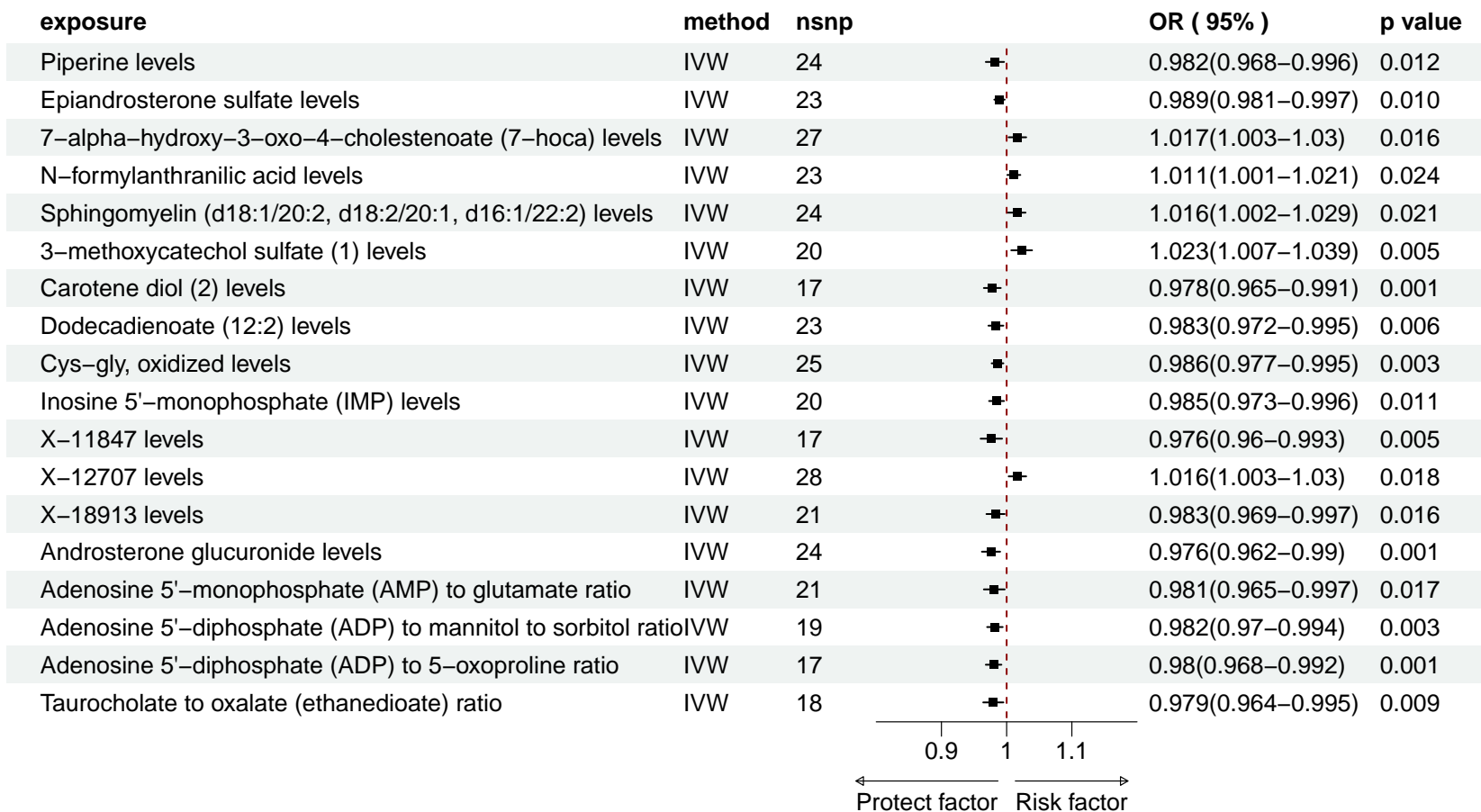

Supplement: Multimedia component 5 [file mmc5.pdf]

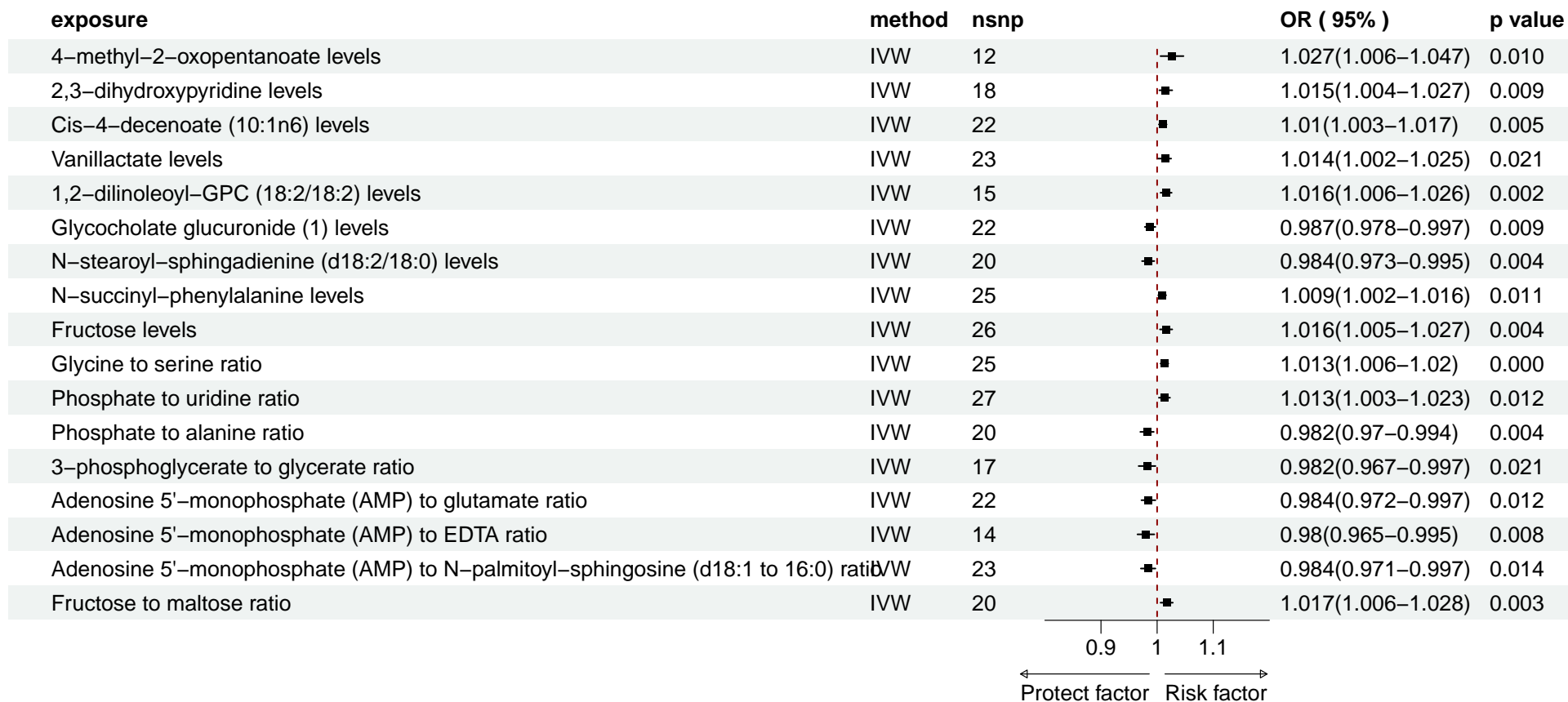

Supplement: Multimedia component 7 [file mmc7.pdf]

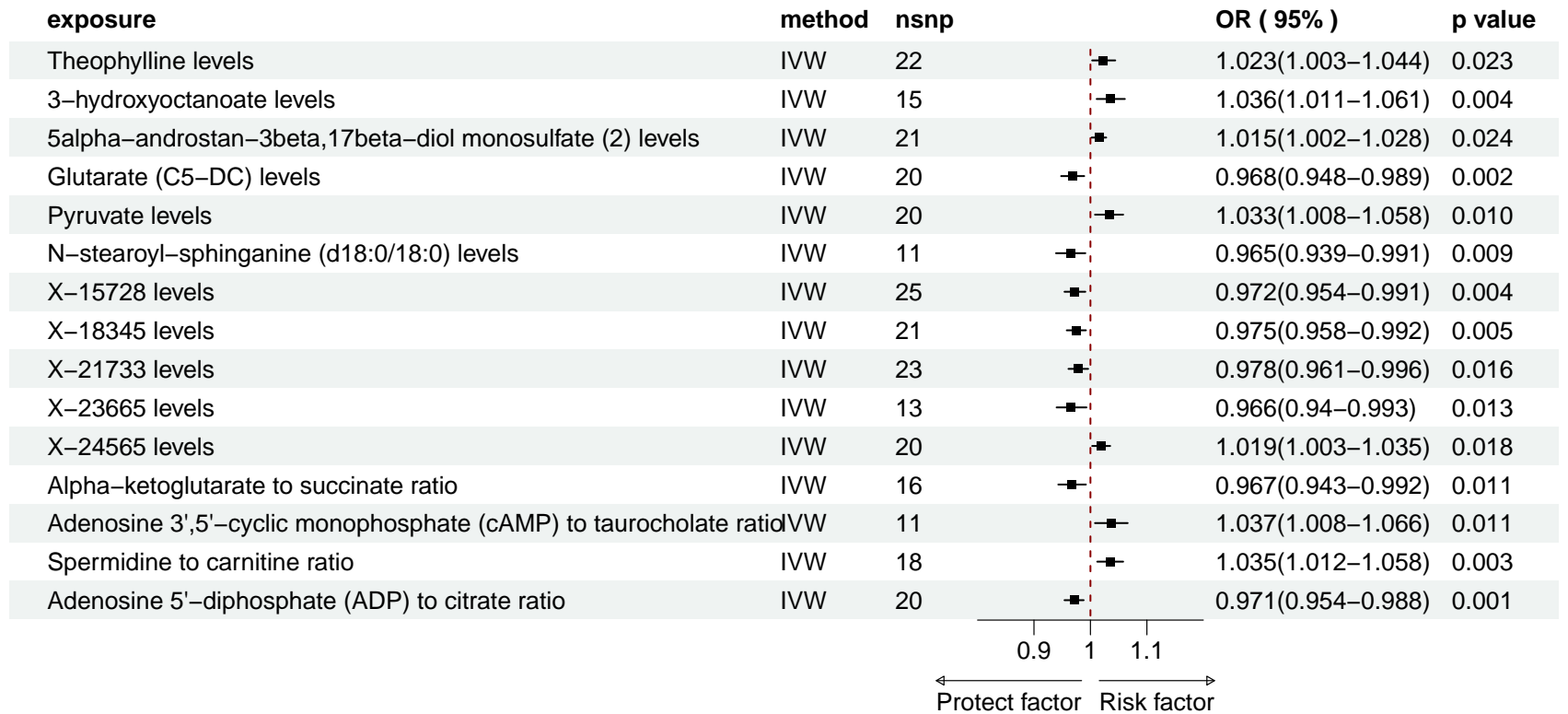

Supplement: Multimedia component 8 [file mmc8.pdf]
