# Supplementary material for: Causal relationship between plasma metabolites and chronic regional pain: a Mendelian randomization study
Source: Metabol Open. 2026 Mar 7;30:100456. doi: 10.1016/j.metop.2026.100456 (PMC12996675; doi:10.1016/j.metop.2026.100456)

exposure	method	nsnp	OR (95%)	p value
Maltotriose levels	IVW	24	0.988(0.979−0.998)	0.013
Palmitoylcarnitine levels (Metabolon platform)	IVW	29	0.984(0.973−0.995)	0.004
Decanoylcarnitine (C10) levels	IVW	25	0.988(0.978−0.997)	0.013
7−methylxanthine levels	IVW	19	1.019(1.004−1.033)	0.010
5alpha−pregnan−3beta,20alpha−diol monosulfate (2) levels	IVW	29	1.013(1.003−1.023)	0.012
Indole−3−carboxylate levels	IVW	13	0.982(0.968−0.995)	0.009
1−(1−enyl−palmitoyl)−2−linoleoyl−GPE (p−16:0/18:2) levels	IVW	21	1.016(1.004−1.029)	0.009
Palmitoleoylcarnitine (C16:1) levels	IVW	19	0.982(0.967−0.997)	0.020
Cortolone glucuronide (1) levels	IVW	24	0.985(0.974−0.997)	0.015
Oleoyl ethanolamide levels	IVW	25	0.985(0.973−0.997)	0.016
Alpha−ketobutyrate levels	IVW	15	0.976(0.958−0.994)	0.010
Arachidonate (20:4n6) to oleate to vaccenate (18:1) ratio	IVW	17	0.987(0.978−0.996)	0.003
Phosphate to mannose ratio	IVW	24	1.014(1.002−1.025)	0.020
Spermidine to (N(1) + N(8))−acetylspermidine ratio	IVW	22	1.019(1.005−1.033)	0.007
Phosphate to N−palmitoyl−sphingosine (d18:1 to 16:0) ratio	IVW	23	0.982(0.97−0.995)	0.007
Phosphate to glutamine ratio	IVW	26	1.018(1.005−1.03)	0.005
Threonine to pyruvate ratio	IVW	30	0.985(0.973−0.997)	0.017

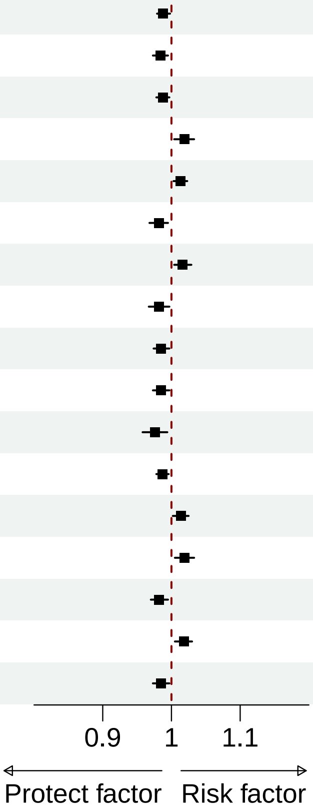

Supplement: Multimedia component 4 [file mmc4.pdf]
