# Supplementary material for: Causal relationship between plasma metabolites and chronic regional pain: a Mendelian randomization study
Source: Metabol Open. 2026 Mar 7;30:100456. doi: 10.1016/j.metop.2026.100456 (PMC12996675; doi:10.1016/j.metop.2026.100456)

exposure	method	nsnp	OR (95%)	p value
1,3−dimethylurate levels	IVW	25	0.988(0.979−0.998)	0.013
Docosapentaenoate n3 DPA; 22:5n3 levels	IVW	25	1.015(1.005−1.025)	0.004
Phenol sulfate levels	IVW	20	1.015(1.003−1.026)	0.012
Taurocholenate sulfate levels	IVW	35	0.989(0.982−0.997)	0.005
N−oleoyltaurine levels	IVW	20	0.986(0.977−0.995)	0.003
3−hydroxypyridine sulfate levels	IVW	21	1.016(1.003−1.029)	0.018
1−(1−enyl−palmitoyl)−2−arachidonoyl−GPE (p−16:0/20:4) levels	IVW	21	1.018(1.007−1.028)	0.001
Sphingomyelin (d18:0/18:0, d19:0/17:0) levels	IVW	26	1.014(1.004−1.023)	0.004
Nisinate (24:6n3) levels	IVW	18	1.011(1.002−1.019)	0.018
Sulfate of piperine metabolite C16H19NO3 (3) levels	IVW	26	1.015(1.005−1.024)	0.003
N−formylmethionine levels	IVW	20	0.986(0.975−0.996)	0.006
Pentadecanoate (15:0) levels	IVW	21	1.016(1.004−1.027)	0.009
X−17335 levels	IVW	14	0.982(0.968−0.996)	0.013
X−25810 levels	IVW	33	0.988(0.98−0.996)	0.005
Adenosine 5'−monophosphate (AMP) to proline ratio	IVW	19	0.984(0.973−0.995)	0.005
Creatine to carnitine ratio	IVW	19	1.014(1.003−1.025)	0.015
Adenosine 5'−monophosphate (AMP) to valine ratio	IVW	20	0.983(0.972−0.995)	0.004

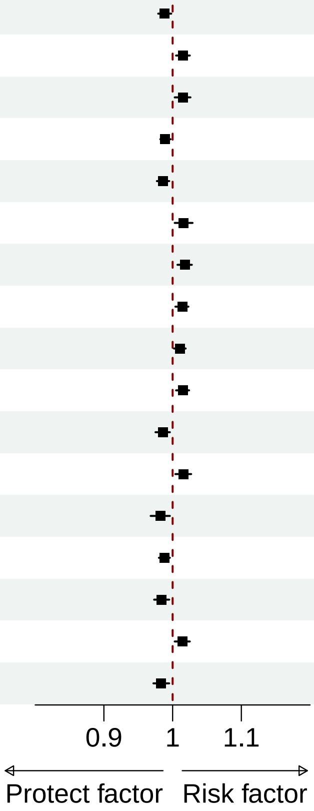

Supplement: Multimedia component 6 [file mmc6.pdf]
